# Fostering pupils’ critical health literacy: examining the potential of physical education in lower secondary school

**DOI:** 10.3389/fspor.2023.1205716

**Published:** 2023-06-13

**Authors:** Anders L. Hage Haugen, Kirsti Riiser, Marc Esser-Noethlichs, Ove Edvard Hatlevik

**Affiliations:** ^1^ Faculty of Teacher Education and International Studies, Oslo Metropolitan University, Oslo, Norway; ^2^ Faculty of Health Science, Oslo Metropolitan University, Oslo, Norway; ^3^ Faculty of International Studies and Teacher Education, Oslo Metropolitan University, Oslo, Norway

**Keywords:** physical education (P.E.), critical health literacy, health, lower secondary school, adolescents, quantitative research approaches

## Abstract

**Background:**

In Norway, the introduction of an interdisciplinary subject named Public Health and Life skills has brought about renewed attention to how health is conceptualized and taught within and across school subjects. Physical education (PE) is one subject that has traditionally been linked to health outcomes. However, a narrow focus on increased physical activity as the main outcome of PE could be counterproductive in the pursuit of health. Critical health literacy (CHL) is put forward as a resource for health that can be nurtured in the PE context; this study hypothesizes that academic achievement in PE is positively associated with some aspects of CHL.

**Methods:**

This cross-sectional study included 521 pupils aged 13–15 years old from five lower secondary schools in Norway. Structural equation models were used as the primary statistical analysis to test the hypothesis. The study controlled for parents’ education, leisure physical activity, and participation in sports club activities.

**Results:**

The results confirm the hypothesis, showing a positive and significant association between PE and CHL. The association remains when controlling for parents’ education, leisure physical activity, and participation in sports club activities (${\beta \;\;}^{\hat{\;}}$_PE_→_CHL−C1 _= 0.264, *p* = 0.001; ${\beta \;\;}^{\hat{\;}}$_PE_→_CHL−C2 _= 0.351, *p* < 0.000).

**Conclusion:**

In our sample, academic achievement in PE was associated with higher levels of CHL. This study contributes to the ongoing discussion on the health benefits of PE. We argue that a resource-based health perspective can produce the appropriate aims for health in PE contexts and that the CHL concept contributes to illuminating key areas, promoting suitable teaching strategies, and bringing balance between an individual and collective focus for future health education, both within PE and across different subjects in school contexts.

## 1. Introduction

Public health and life skills (HLS) was introduced as an interdisciplinary topic in the Norwegian Core Curriculum (1). Traditionally, in Norway, education about health has been distributed to different subject areas, such as physical education (PE), food and health, and science. Although the new Core Curriculum elevates health as an interdisciplinary topic, the National Curriculum only describes the competence aims for specific subjects. The degree to which these subject programs and competence aims connect to HLS vary (2). Therefore, it is the school owners, principals, and teachers’ responsibility to operationalize HLS within and across different subjects.

PE has traditionally been legitimized, in part, by its emphasis on promoting health and fitness. This sets it apart from many other school subjects, which may prioritize academic or vocational skills. This focus on health has been recognized as a key attribute of PE both in Norway (3) and internationally (4). In a recent review of the research on the didactics of health in PE, the authors identified two broad categories of studies based on different health perspectives: (I) biomedical and (II) resource-based health perspectives (5). The former category of studies has tended to emphasize maximizing levels of moderate to vigorous physical activity (PA). In the latter category, health is positioned as an object of learning, and as a result, a variety of resources for health (e.g., social abilities, critical reflection) are put forward. An interesting finding is that most empirical research is done within the former category; consequently, empirical knowledge from resource-based health perspectives in PE is scarce (5). In the present study we position health as a learning object and propose critical health literacy (CHL) as an individual and collective resource for health that can be nurtured within PE. More precisely, we examine the associations between academic achievement in the school subject PE and elements of CHL. To build a rationale for this investigation, we first take a closer look at how health has been conceptualized and taught within PE and why health perspectives seem to matter. We then present the concept of CHL and its utility as a framework for education about health in school and PE.

### 1.1. Physical education and health

In the biomedical approach to health in PE, the benefits of PE have largely been linked to improving or maintaining population physical fitness and/or hygiene (3, 6). The consideration of children's varying physical abilities has contributed to a gradual shift from emphasizing physical fitness to just increasing PA. At the turn of the millennium, there was a growing public concern for physical inactivity, sedentary behavior, and the rise of noncommunicable conditions such as obesity and type II diabetes. This contributed to a call from professionals and politicians alike for more PA in schools in general (7) and in PE specifically (8, 9). In Norway, the advocates of PE's public health potential even went as far as to explicitly state that health, represented by increased energy expenditure, was the only relevant goal of PE and that this had to be prioritized before any pedagogical concerns about learning a curricula (10). Following this line of argument, the health benefits of PE have mainly been linked to the cumulative amount of PA it produces in its students (e.g. 11–16). Several scholars have critiqued the usefulness of a biomedical health perspective for the PE context (e.g. 8, 17, 18); they warned against how this might lead to adopting a healthism ideology (19, 20), which situates the problem and solutions of health and disease at the level of the individual. Such approaches could end up rewarding the athletic and those who are already engaging in regular PA and sports activities, while the less athletic and those who are not so active are put at a disadvantage. This is reflected in the Norwegian PE context, where it is the sports-active youth who seem to collect the most benefits from the subject (21). In particular, girls who do not participate in sports seem to benefit the least from PE (22). Importantly, well-intended programs for health promotion through PE that emphasize increased PA could end up being detrimental to some students’ health and well-being (18), if they are not sensitive to the various needs related to adolescents background and previous experiences. In line with this critique, it has been argued that health should be viewed as a ‘learning object’ within educative contexts. Quennerstedt (23) made a case for a resource-based health perspective, in which attention can be drawn to the abilities, knowledge, and skills that pupils should develop through education, rather than merely increased levels of PA, which characterizes a biomedical health perspective in PE. Mong and Standal (5) found that, in most such programs, teaching strategies were primarily based on instructive methods of predetermined content. On the other hand, among resource-based or critical approaches, there has been a greater tendency toward recommending non-instructive and participatory strategies, in which teachers, together with the students, become the facilitators of learning.

Shifting the focus from maximizing PA to learning for health through participatory methods may be a beneficial transition in PE. However, this shift does not necessarily address the criticism of healthism: too much responsibility for one's health is placed on the individual. Therefore, it is pivotal to balance the focus on individual skills for health with an emphasis on the collective attributes for health and well-being. In PE, such abilities can include appreciating and supporting the needs of others, as well as participating in the democratic processes and decisions that concern the collective.

### 1.2. Critical health literacy

CHL provides a framework for thinking about health as both an individual and collective matter (24). In line with the resource-based health perspective, the concept has been put forward and promoted to avoid the pitfalls of traditional health education efforts that are dominated by the instruction and delivery of predetermined content (25, 26). The concept has roots within emancipatory critical pedagogy (27–29) and consists of three overlapping and interconnected domains: information appraisal, understanding the social determinants of health, and the abilities that enable actions that can promote health and well-being in a collective (30, 31). The first domain of information appraisal is a complex cognitive skill that is essential for pupils to gain agency for health and well-being (32, 33); it includes abilities and strategies to judge the credibility and relevance of health-related information (31). The majority of CHL research revolves around these cognitive abilities, both in general public health research (24, 30, 34) and in school contexts (35). The second domain partially depends on the cognitive skills of appraising and understanding health-related information in its proper context, but it also borders on affective and attitudinal abilities (36). The domain involves an appreciation of how individuals have different opportunities for health and, subsequently, that there is a balance between individual and collective responsibility for health. Finally, the third domain revolves around the ability to act to the benefit of one's own and others’ health and well-being, and these actions inevitably depend on personal and social abilities (30). In a recently developed measurement framework for CHL in school contexts, these abilities are operationalized as social actions that adolescents can take to become active agents for health and well-being in a collective (31, 36).

### 1.3. Aim and purpose

As most of the empirical health-related research in PE tend to derive from a biomedical understanding of health (5) we aim to shed light on alternative ways in which PE can contribute to overall education about health in schools, by examining the relationship between academic achievement in PE and CHL. Although PE might be a suitable context for nurturing all aspects of CHL, we have chosen to focus on the third domain in the present study because PE is an appropriate context for developing social interaction abilities (37). The subject qualitatively differs from regular classroom settings by providing a more dynamic and interactive learning environment (38). In addition, the new national curriculum for PE highlights participation and cooperation as one of three core elements of the subject. Abilities to recognize differences, include others and reflect around equality are emphasized (1). Therefore, there are reasons to believe that CHL-C abilities can be nurtured within the PE context. Because achievement in PE are unequally distributed in various groups of students, we control for parents’ education, participation in sports club activities, and leisure PA. The following hypothesis is tested: Academic achievement in PE is positively associated with levels of CHL, and the relationship remains when controlling for parents’ education, leisure PA, and participation in sport club activities.

## 2. Materials and methods

### 2.1. Sample

This cross-sectional study was part of the Literacies for Health and Life Skills project at Oslo Metropolitan University (39) and was conducted in five partner schools during the autumn of 2021. All the lower secondary schools were in medium-income municipalities in the area surrounding the capital of Norway, and they varied in size, with two small schools (< 200 pupils), one medium-sized school (250–350 pupils), and two large schools (> 450 pupils). Approximately 1,592 pupils aged 13–15 years old attended the schools at the time of data collection. After school management agreed to participate, two video presentations with standardized information for the pupils were sent to the schools. The teachers were instructed to use the first one to introduce the study before handing out written consent forms to pupils and their parents. The second provided standardized information to be presented shortly before conducting the survey, repeating the main purpose of the study, and instructing about the practicalities. In total, 522 pupils consented and responded to the digital survey during school hours. Those who did not provide written consent from their guardians were given alternative assignments.

### 2.2. Measures

CHL was measured with the two scales from the Critical Health Literacy for Adolescents Questionnaire (CHLA-Q) (31, 36) that specifically target the third domain of CHL. Both scales consist of three indicators measuring perceived abilities to support others (CHL-C1) and perceived abilities to participate in discussions regarding health (CHL-C2). The indicators are measured on a five-point Likert scale (1: completely incorrect; 2: incorrect; 3: sometimes correct; 4: correct; 5: completely correct), and each item starts with the phrase: “I am a person who…” (chl36: …can help others if they are not doing well; chl37: …can contribute to the well-being of others in my class; chl38: …can help find solutions that are acceptable to all parties; chl39: …can easily talk to others, even if I don't know them very well; chl40: …can share information with others about factors that influence health; chl41: …believe my knowledge about health could be useful for others). Descriptive statistics for each indicator are presented in Supplementary Materials. Performance in PE was measured using one indicator asking pupils which grades (1–6) they expected to achieve in PE this semester. Leisure PA was measured with one question: “Outside of school hours, how many times during a week do you participate in PA, to the extent that you become out of breath and/or begin to sweat?” (1: 0–1 time; 2: 2–3 times; 3: 4–5 times; 4: more than 5 times). Participation in sports club activities was measured with one item: “Are you an active member of a sports club? (e.g., football, handball, cross-country skiing, tennis, gymnastics, athletics, swimming)” (1: never been a member; 2: no, but I have been a member previously; 3: yes). The indicators of PA and SPORT were inspired by the national survey study of PA in Norway (40). Parents’ education (ParEd) was initially measured using two items: one for mothers’ education and one for fathers’ education. Both items were formulated in the same way: “mothers/fathers’ education?” (1: Higher education [university or college], 2: High school/upper secondary school; 999: don't know/doesn't want to answer). In the final data analysis, we combined the responses for the parents’ education (0: only 1 or none of parents have higher education/don't know; 1: both parents have higher education), participation in sports club activities (0: not active; 1: active member), and PA (1: > 3 times, 0: < 4 times) into two categories.

### 2.3. Data analysis

All statistical analyses were performed using RStudio (RStudio Team, version 2021.9.0.351). The “tidyverse” packages (41) was used for data preparation, and the “lavaan” package (42) was used for confirmatory factor analysis (CFA) and structural equation model (SEM) analysis. Following recommendations for SEM analysis with Likert-type items of five categories or less, all variables in our study were treated as ordinal (43, 44), and the means and variance adjusted unweighted least square (ULSMV) estimator was applied (45–47).

Initially, we examined mean group differences in gender, grades, ParEd, leisure PA, and SPORT for the outcome variables academic achievement in PE, CHL-C1, and CHL-C2. The independent sample t-test was used in cases with two groups, and an ANOVA test was used to test for statistically significant differences between grades.

#### 2.3.1. SEM analysis

In the SEM model, academic achievement in PE was positioned as a predictor of CHL-C, and we measured this with only one indicator: expected grade in PE. Assuming that the predictors are free of measurement error is generally not recommended in SEM analysis, especially if we know that some error is likely (43). Therefore, we chose to specify academic achievement in PE as a latent variable for which measurement errors can be manually set, despite the uncertainty related to quantifying the precision with which the construct is measured. We decided to specify a factor loading (λ) of 0.80, meaning that academic achievement in PE (as a latent variable) explained 64% of the variation in the observed indicator of expected grade. In the Supplementary materials, we show how model parameters change for different levels of measurement precision specified (λ = 0.70; λ = 0.90). The relatively small changes provided justification for the specification of the factor loading.

**Figure 1:**
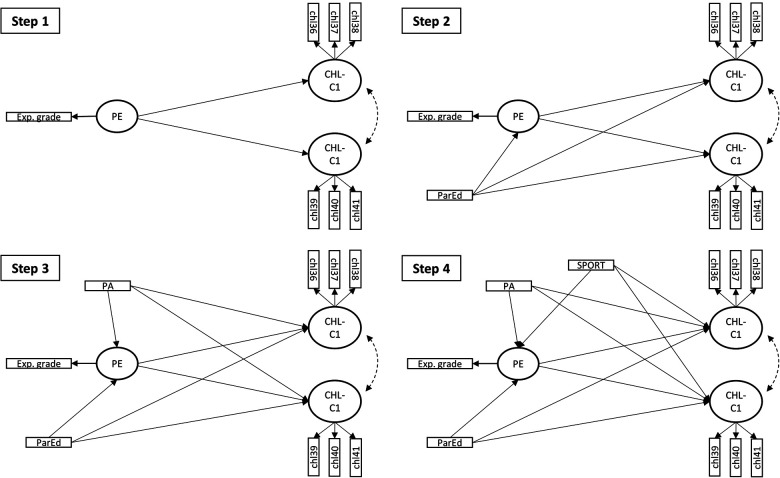
Steps in the SEM analysis. Exp grade, Expected grade in physical education; PE, Physical education; CHL-C1, Critical Health Literacy scale C1; CHL-C2, Critical Health Literacy scale C2; PA, Leisure physical activity; SPORT, Participation in sport club activities; ParEd, Parents’ level of education.

Further analysis was performed in four main steps. As presented in Figure 1, each step introduced a new parameter to the model. The global and local model fit was evaluated for each step. We report recommended fit indices such as chi-square test (χ^2^_mvadjusted_) of exact fit (*p* ≥ 0.05), comparative fit index (CFI ≥ 0.95), root mean square error of approximation (RMSEA ≤ 0.05), and standardized root mean residuals (SRMR ≤ 0.05). Before we added regressions to the model, we examined the closeness of fit for the measurement models. We emphasize the unbiased standardized root mean residuals adjusted for average communality (uSRMR/$\bar{R}$ ^2^ ≤ 0.5), along with a criterion of no individual residuals above 0.1 (48, 49), aligning with the original study (36). This statistic is only available for the measurement model, so we only report traditional fit indices for the remaining stages of analysis, in addition to an examination of individual residuals.

In the first step, we added a regression path from PE to CHL-C1 and CHL-C2. In this step, we evaluated the closeness of model fit with uSRMR/$\bar{R}$ ^2^ along with a criterion of no individual residuals above 0.1 for close fit. In the subsequent steps, we added regression parameters to the model and monitored how the model parameters and fit changed for each step. In the second step, ParEd was added to the model with a regression path to PE and both CHL scales; thereafter, we added PA and SPORT by regressing CHL-C1, CHL-C2, and PE on these variables. In Supplementary materials we provide a step-by-step overview of model syntax and the commands used. We also provide tables with the residuals for each step in the analysis.

## 3. Results

### 3.1. Descriptive results and group differences

A total of 521 adolescents responded to the full survey, which yielded a response rate of 33% when calculated as responses from the total eligible population. On average, it took 17 min to complete the full survey. Table 1 shows that approximately the same number of boys and girls participated. Two-thirds (total = 66.8%, boys = 70%, girls = 63%) of the sample were active members of sport clubs, and 40% reported that both parents had more than three years of higher education. Almost half the sample (total = 49%, boys = 58%, girls = 41%) reported being physically active three times or more during a week, outside of school hours. The overall mean of the expected grades in PE was 5.0, while the mean ranged from 4.5 to 5.3 in the various group variables (gender, grade, parents’ education, member of sport clubs and level of PA). For CHL-C1, the means ranged from 11.3 to 12.1, with the total mean at 11.7. The CHL-C2 sample means were slightly lower at 9.7, with group means ranging from 9.2 to 10.2. Those who were active members of sports clubs and reported being physically active four times or more during a week scored significantly higher than their peers in all outcome variables. This was also the case for pupils who reported that both parents had more than three years of higher education. Boys scored significantly higher than girls on the expected grades in PE and CHL-C2 scales; however, girls scored higher on the CHL-C1 scale, though the difference was not significant. There were no significant differences between grades.

**Table 1:** Sample characteristics and descriptive statistics.

Variables		PE	CHL-C1	CHL-C2
	*N* (%)	Mean (SD)	Mean (SD)	Mean (SD)
**Total**	521	5.0 (0.9)	11.7 (2.1)	9.7 (2.8)
Gender				
Boys	248 (47.6)	5.2 (0.8)	11.5 (2.2)	10.0 (2.7)
Girls	273 (52.4)	4.8 (0.9)***	11.9 (2.0) NS	9.5 (2.9) NS
Grade				
8th grade	164 (31.5)	5.0 (0.9)	11.7 (2.1)	9.4 (2.8)
9th grade	190 (36.5)	4.9 (0.8)	11.6 (2.1)	9.9 (2.8)
10th grade	167 (32.1)	5.1 (1.0) NS	11.8 (2.1) NS	9.8 (2.6) NS
Parents education				
Both > 3 years	210 (40.3)	5.2 (0.8)	11.9 (1.9)	10.1 (2.7)
One or both < 3 years/don't know	311 (59.7)	4.9 (1.0)***	11.6 (2.2)*	9.5 (2.8)*
Participation in sports club activities				
Active member	347 (66.8)	5.2 (0.7)	11.9 (1.9)	9.9 (2.7)
Not active member/never been member	174 (33.4)	4.5 (0.9)***	11.3 (2.3)**	9.3 (2.9)*
Leisure physical activity				
4 times or more during a week	255 (48.9)	5.3 (0.7)	12.1 (1.9)	10.2 (2.6)
3 times or less during a week	266 (51.1)	4.7 (0.9)***	11.4 (2.2)***	9.2 (2.9)***

PE was measured as expected grade in the present semester on a scale from 1 to 6. Sum scores in the CHL scales ranged from 3 to 15. Differences between dichotomous groups were tested for significance using an independent sample *t*-test. An ANOVA test was used to test for statistically significant differences between grades.

NS *p* ≥ .05.

* *p* < .05.

** *p* < .005.

*** *p* < .000.

### 3.2. Results from the SEM analysis

Before performing the SEM analysis, we estimated a measurement model with the latent variables (Table 2). Covariance was estimated freely between all latent variables. The factor loadings were strong, ranging from 0.699 to 0.846 and significant (*p* < 0.000). The chi-square test was significant; however, the residuals of the model were small, as reflected with the uSRMR = 0.032. When adjusting for the average communality, the model also displayed a close fit (uSRMR/$\bar{R}$ ^2^**^ ^= **0.052). No individual residuals were above 0.10. Thus, we retained the model and continued with stepwise SEM analysis.

**Table 2:** Confirmatory factor analysis of latent variables (first step of the analysis). Standardized and unstandardized factor loadings with standard error (SE) and *p*-values.

Parameter	Unstandardized		Standardized		
	Estimate	SE	Estimate	SE	*P*-value
CHL-C1					
chl36	0.776	0.026	0.776	0.026	0.000
chl37	0.846	0.024	0.846	0.023	0.000
chl38	0.823	0.025	0.823	0.024	0.000
CHL-C2					
chl39	0.699	0.030	0.699	0.031	0.000
chl40	0.787	0.026	0.787	0.027	0.000
chl41	0.766	0.026	0.766	0.027	0.000
PE					
Exp. Grade	0.800	—	0.800	0.007	—

χ ^2^_mvadjusted _= 52.303 (df = 12, *p* < 0.000), CFI = 0.978, RMSEA = 0.080 [0.059, 0.103], SRMR = 0.036, uSRMR = 0.032 [0.023, 0.042], uSRMR/$\bar{R}$ ^2^ **= **0.052.

In the first step we added regression paths from PE to CHL-C1 and CHL-C2. We found a significant relationship between the predictor (PE) and outcome variables CHL-C1 and CHL-C2 (Step 1: ${\beta \;\;}^{\hat{\;}}$_PE_→_CHL−C1 _= 0.310, *p* = 0.000, ${\beta \;\;}^{\hat{\;}}$_PE_→_CHL−C2 _= 0.368, *p* = 0.000). In the second step, we added parents’ education (ParEd) to the model as a predictor of PE and both CHL scales. ParEd had a significant direct effect on PE (${\beta \;\;}^{\hat{\;}}$_ParEd_→_PE _= 0.217, *p* = 0.000) but not on the CHL scales; the relationship between PE and CHL became, as expected, slightly weaker, though negligibly so (Step 2: ${\beta \;\;}^{\hat{\;}}$_PE_→_CHL−C1 _= 0.301, *p* = 0.000, ${\beta \;\;}^{\hat{\;}}$_PE_→_CHL−C2 _= 0.353, *p* = 0.000). The fit indices improved as we added complexity to the model (χ^2^_mvadjusted _= 29.646 (df = 16, *p* < 0.020), CFI = 0.992, RMSEA = 0.040 [0.016, 0.063], SRMR = 0.038). In step 3, we repeated the procedure from the previous step, but now, we added PA to the model as well. The relationship between PE and the CHL scales became somewhat weaker because the additional variables had their own direct paths to the CHL scales; however, the reduction was relatively small (Step 3: ${\beta \;\;}^{\hat{\;}}$_PE_→_CHL−C1 _= 0.268, *p* = 0.000, ${\beta \;\;}^{\hat{\;}}$_PE_→_CHL−C2 _= 0.326, *p* = 0.000). PA had a stronger effect on PE than ParEd (Step 3: ${\beta \;\;}^{\hat{\;}}$_PA_→_PE _= 0.441, *p* = 0.000, ${\beta \;\;}^{\hat{\;}}$_ParEd_→_PE _= 0.130, *p* = 0.020). None of the added variables had a significant direct effect on CHL. Model fit did not change significantly between step 2 and step 3 (step 3: χ^2^_mvadjusted _= 36.585 (df = 20, *p* < 0.013), CFI = 0.990, RMSEA = 0.040 [0.018, 0.060], SRMR = 0.037). Following the same procedure in the final step, we added participation in sports clubs to the model. The relationship between the PE and CHL scales remained approximately the same (Step 4: ${\beta \;\;}^{\hat{\;}}$_PE_→_CHL−C1 _= 0.264, *p* = 0.001, ${\beta \;\;}^{\hat{\;}}$_PE_→_CHL−C2 _= 0.351, *p* = 0.000). The three background variables all had a significant and positive effect on PE (Step 4: ${\beta \;\;}^{\hat{\;}}$_ParEd_→_PE _= 0.108, *p* = 0.045, ${\beta \;\;}^{\hat{\;}}$_PA_→_PE _= 0.257, *p* = 0.000, ${\beta \;\;}^{\hat{\;}}$_SPORT_→_PE _= 0.332, *p* = 0.000). None of the background variables had a significant direct effect on CHL in this model.

**Figure 2:**
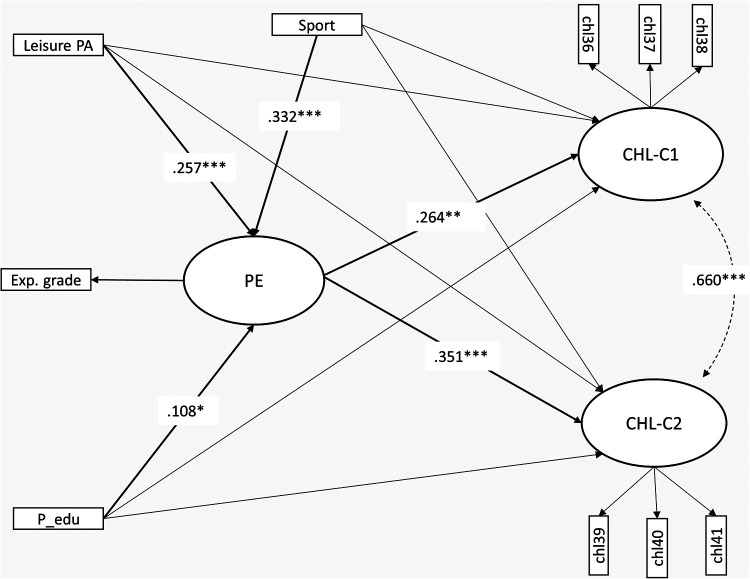
Partially latent structural regression model of relationships between leisure physical activity (PA), participation in sport club activities (SPORT), parents education (parEd), academic achievement in physical education (PE), and the third domain of critical health literacy (CHL-C1, CHL-C2). Model fit: χ^2^_MV _= 31.156, df = 24, *p* = .149, RMSEA = 0.024 [0.000–0.045], CFI = 0.996, SRMR = 0.036. Estimator: ULSMV. *** *p* < .000, ** *p* < .005, * *p* < .05. Nonsignificant relationships are suppressed.

The final model (Figure 2) displayed an excellent model fit (χ^2^_mvadjusted _= 31.156 (df = 24, *p* < 0.149), CFI = 0.996, RMSEA = 0.024 [0.000, 0.045], SRMR = 0.036). In Table 3, we present the results from the regressions in the model. We found a positive and significant effect from PE on both the dependent variables CHL-C1 (${\beta \;\;}^{\hat{\;}}$_PE_→_CHL−C1 _= 0.264, *p* = 0.001) and CHL-C2 (${\beta \;\;}^{\hat{\;}}$_PE_→_CHL−C2 _= 0.351, *p* < 0.000). None of the background variables had a significant direct effect on the dependent variables; however, they all had a significant and positive effect on PE, in total explaining 30.1% of the variance in PE. Altogether, the direct and indirect effects in the model explained 8.9% and 13.1% of the variation in CHL-C1 and CHL-C2, respectively. The majority of this came from the direct effect of PE, as can be derived by squaring the standardized regression coefficients (R^2^_PE_→_CHL−C1_ = 0.070, R^2^_PE_→_CHL−C2_ = 0.123).

**Table 3:** Standardized and unstandardized regression coefficients with standard errors (SE), *p*-value. R-squared is given for dependent variables.

	Unstandardized		Standardized			
Parameter	Estimate	SE	Estimate (${\beta \;\;}^{\hat{\;}}$)	SE	*P*-value	R-squared
**CHL-C1**						8.9%
* PE*	0.231	0.068	0.264	0.074	0.001	—
* Parents ed*.	0.063	0.111	0.029	0.052	0.572	—
* Leisure PA*	0.131	0.131	0.063	0.062	0.314	—
* Sport clubs*	-0.029	0.141	-0.013	0.063	0.837	—
**CHL-C2**						13.1%
* PE*	0.315	0.075	0.351	0.077	0.000	—
* Parents ed*.	0.118	0.113	0.054	0.052	0.297	—
* *Leisure PA	0.179	0.132	0.083	0.061	0.174	—
* *Sport clubs	-0.258	0.143	-0.114	0.062	0.072	—
**PE**						30.1%
* Parents ed*.	0.262	0.131	0.108	0.053	0.045	—
* *Leisure PA	0.614	0.144	0.257	0.058	0.000	—
* *Sport clubs	0.840	0.153	0.332	0.056	0.000	—

## 4. Discussion

In Norway the introduction of the interdisciplinary subject HLS has brought about renewed attention to education about health. The present paper has addressed how variation in students’ CHL-C can be explained by their academic achievement in PE, when controlling for the independent variables of parents’ education, participation in sports, and leisure PA. In the initial analysis, we examined how the means of the dependent variables of expected grade in PE, CHL-C1, and CHL-C2 were distributed across groups in our sample. Those who participated in sports club activities and reported being physically active more than three times during a week scored significantly higher than their peers in PE and on both CHL-C scales. This aligns with previous research from Finland which found that sport club participation were associated with higher levels of perceived HL (50). Previous research in the Norwegian PE context has found that youth's participation in organized sports is important for positive attitudes toward PE (21), and international and Scandinavian researchers alike have pointed out the dominant position of competitive and performance-based sports culture in PE contexts (21, 22, 51, 52). Although sport activities are part of the Norwegian curricula, the high amount of this type of activity reported by adolescents in Norway seems to be disproportionate, thus leading to a subject favoring a certain type of pupils (22). In line with national end-term grades (53) there were also significant differences between boys and girls of our sample in academic achievement in PE. There was also a slightly larger proportion of boys who participated in sport club activities and who reported leisure PA more than three times during a week. This pattern corresponds well with objectively measured PA in Norway (54). Those who reported having two parents with more than three years of higher education scored higher on PE and both CHL-C scales. Because parents’ education is a known marker of socioeconomic status, this was expected.

In the SEM analysis, we tested the hypothesis of a positive association between success in PE and CHL-C abilities and if this relationship remained when we added parent's education, leisure PA, and participation in sport club activities to the model. We positioned participation in sport club activities, leisure PA, and parents’ education as the predictors of success in PE. These variables explained a substantial amount of variation in PE in our study, aligning well with both theoretical arguments proposed by PE scholars (e.g., 52) and empirical research (21, 22). Similarly, PE is positioned to explain variance in the CHL-C scales, and the overall model showed a moderate positive association between PE and perceived abilities to participate in discussions regarding health (CHL-C2) and a slightly weaker positive association between PE and perceived abilities to provide social support (CHL-C1). The fact that the relationship between success in PE and CHL-C remained when sport participation and leisure PA were controlled for can be interpreted as an indication of PE's potential for developing students’ CHL-C abilities. If PE teachers take the opportunity to enhance the development of CHL-C abilities in all students, this could help counteract health inequities. In our sample, the association between performance in PE and CHL-C is not dependent on participation in sports or leisure physical activity, although it should be noted that the relationship is not particularly strong. Therefore, caution is needed when making claims about the practical importance of these results, and it is important to acknowledge that we cannot determine causality from cross-sectional data alone. The associations found in our study could suggest that CHL-C abilities are learned in PE, or that abilities acquired elsewhere are rewarded with higher grades in PE teacher assessments. Both explanations suggest that fostering CHL-C abilities in PE is possible, if we assume that students to some degree act in accordance with what they perceive is valued by their teachers. However, these results can also be interpreted through a more critical lens. If students who display confidence in their abilities are rewarded with higher grades, this may marginalize those who are less confident. It is crucial that teachers strive to include all students in decision-making and content negotiation regarding education about health and well-being in PE. Teachers must facilitate the development of CHL-C abilities in all students and, ensure that these abilities are not merely distributed among high-achieving students in the subject.

Importantly, learning does not automatically occur as a result of participating in PA or doing sports within PE, but depends on contextual and pedagogical considerations (55). Increasing students’ agency and abilities to participate as active citizens for health and well-being is pivotal to the CHL concept (36, 56), and this arguably calls for student-active approaches that encourage action, reflection, and democratic values (57). It should be noted that, in the current field of PE, from which our sample was drawn, there is most likely a variety of health understandings and didactical approaches practiced. Also, various related social abilities have long been important learning objects in Norwegian PE curricula (58), and there are several standards in the new PE curriculum that revolve around social competencies that are relatable to CHL abilities (1). Until the recent renewal of the curricula, the concept of “fair play” was explicitly emphasized in the curriculum. Important aspects of fair play, as operationalized in the previous curriculum, were to support others, contribute to collaboration, and acknowledge differences (e.g., different opportunities for participation) (59). Furthermore, international traditions within PE and sports tend to emphasize the importance of these arenas for the promotion of social abilities (37, 38, 60). These objects of learning all align with CHL-C competencies, as operationalized in the current study.

The magnitude of variance in CHL-C explained by the model seems plausible, and the statistical fit of the model was excellent. This supports the main hypothesis of our study: learning of CHL-C competencies occurs within the PE subject in our sample. However, there are many different variables ranging from personal traits to family and other social learning arenas that are likely to have an impact on these CHL-C abilities as well. Upcoming studies should be designed to assess how contextual and pedagogical considerations impact the fostering of all aspects of CHL within PE. The main contribution of the present study has been to confirm an association between academic achievement in PE and CHL-C. We aimed to explore empirical grounds for supporting the notion that PE can make alternative contributions to health, particularly in relation to the introduction of the interdisciplinary subject of HLS. We hope that our findings will inspire further research into the relationship between CHL and health education in schools. The future success of the PE subject has been linked to its ability to align content and teaching strategies with aims and purposes (61). We argue that a resource-based health perspective brings forth appropriate aims for health in PE contexts and that the CHL concept contributes to illuminating key areas, promoting suitable teaching strategies, and bringing balance between an individual and collective focus for future health education, both within PE and across different subjects in school contexts. While our research gives cause to be optimistic about the potential of PE to nurture CHL, there are several challenges and limitations in our study that must be acknowledged.

### 4.1. Limitations and calls for future research

Participation in the study was based on willingness to take part and depended on parent's consent. This could lead to a skewed selection from the population. Our sample consisted of an approximately equal number of boys and girls, and an equal number of 8th, 9th and 10th graders. The proportion of sport active youth in our sample were 67%, while estimates in other studies vary. A national survey from 2011 found that 61% of 15-year-olds were active members of sport clubs (40), while more recent publications found that 75% participate at some time during their youth (age 13–18 years old) (62). Another nationally representative survey found that 59% of all lower-secondary school students are active in sport clubs in 2021, however the proportion have ranged from 66% to 59% in the years between 2010 and 2022 (63). This means that our sample might be slightly skewed with a higher proportion of sport active youth, however the differences are small.

The stepwise and transparent analysis applied in the present study is a strength. We utilized scales for CHL that have been developed according to scientific standards (31, 36) and followed best practice recommendations for SEM studies and CFA, including appropriate estimation techniques and fit evaluation (43, 44). We could account for measurement error directly in the estimated models, and the excellent fit of the final model supports the underlying hypothesis. However, the cross-sectional design of the study did not allow us to draw conclusions about the causality of the relationships examined. Possibly it is the higher levels of CHL-C that explain academic achievement in PE, although a reciprocal relationship between success in PE and CHL-C abilities seems likely. Also, whether these abilities are acquired through learning in PE or elsewhere cannot be determined with certainty. There will always be variables not included in the model that could influence complex social constructs, such as CHL. The fact that we did not include measures of the first and second domain of CHL in this study could be considered a limitation. It is possible that students who demonstrate confidence in their interactive and democratic abilities may not necessarily possess the ability to critically appraise health-related information. However, we would argue that fostering these abilities is valuable regardless of their connection to cognitive abilities to appraise health information. In a previous study, we found a moderate association between the first and third domain of CHL (36). Future studies should include objective indicators of CHL, particularly in the domain of critical information appraisal (CHL-A), as demonstrating high confidence in one's ability to judge the credibility of information could be interpreted as an indicator of being non-critical.

Our indicator of academic achievement in PE was the expected grade. In practice, certain behaviors and competencies are rewarded with higher grades. Although grades are based on the same national curricula standards in all Norwegian schools, there are local variations in teachers’ assessment competence (64), interpretations of curricula standards, and, subsequently, what teachers emphasize when grading student achievement in PE. In addition, we could not access the actual grades of students; instead, we asked what grade they expected to get in PE the current semester. Although this is an additional weakness, research has shown that self-reported grades are positively correlated with actual grades and that these are often based on past evaluations, along with an optimistic prediction of future results (65). We also compared mean expected grades for the 10th graders in our sample with the population means for six subjects (Mathematics, Norwegian Language Arts, Social studies, Food and Health, Science and PE) (53). In all subjects the expected grades in our sample were slightly higher than the actual national grades, the differences in means ranged from 0.2 in social studies to 0.4 in mathematics and PE. Differences are likely due to optimistic expectations of grades; however, we cannot rule out that our sample are slightly skewed with a higher proportion of high-achieving students than the population in general.

We utilized only self-reported (subjective) indicators of CHL in the present study. There is generally more measurement error associated with self-measurement as opposed to performance-based (objective) indicators (66). The benefits are, among other things, that they are easy to administer and are experienced as less burdensome for participants to complete (67). Also, an important challenge with objective indicators of CHL is the emphasis this puts on cognitive and functional abilities (e.g., reading and understanding text). This was less relevant in a PE context. With self-reported indicators it is easier to capture different dimensions of complex constructs. In addition, it is well documented that self-efficacy can predict behavior (68). Adding objective indicators could have enhanced the validity of our results, but at the time of our study, performance-based indicators for assessing all aspects of adolescents’ CHL were not yet available (31). Recently, a performance-based instrument for HL encompassing all three domains of CHL has been published (69). The instrument showed promising psychometric properties and could be applied along with self-reported indicators developed in the Norwegian school context (e.g., 36) in upcoming studies. Subsequent investigations should also examine how teaching health in PE and other subjects can contribute to facilitating learning in all aspects of CHL, including the appraisal of health-related information and understanding the social structures that impact health opportunities for all.

### 4.2. Conclusion

We have examined the relationship between academic achievement in PE and the third domain of CHL. The results indicated a positive relationship, and the present practice of PE in our sample seems to nurture CHL-C abilities. The present study contributes to the ongoing discussion on the role of health within the school subject of PE and, subsequently, how PE contributes to HLS and health education in general. We have argued that health is an object of learning in PE and have used the concept of CHL to illuminate and empirically investigate some resources for health that are developed within PE. Importantly, CHL calls attention to the collective and individual attributes for health, which can help counteract an individualistic focus in school-based health education within PE and across different subjects. As such, CHL complements a resource-based health perspective, underpinning the need for student-active approaches that enable content negotiation, action, and reflection regarding matters concerning health and well-being.

## Data Availability

Requests to access the datasets should be directed to aluha@oslomet.no.
