# Supplementary material for: Fostering pupils’ critical health literacy: examining the potential of physical education in lower secondary school
Source: Front Sports Act Living. 2023 Jun 13;5:1205716. doi: 10.3389/fspor.2023.1205716 (PMC10294679; doi:10.3389/fspor.2023.1205716)
Supplement: Supplementary file 1 [file Datasheet1.pdf]

Supplementary Material

Fostering pupils' critical health literacy: Examining the potential of physical education in lower secondary school

Haugen, Anders L. Hage*, Riser, K., Esser-Noetlisch, M., Hatlevik, O., E.

*** Correspondence:** Anders L. Hage Haugen: aluha@oslomet.no

1. Supplementary Data

In this document we provide supplementary data that increase the transparency of the data analysis process in this research. In the first section we show model syntax and commands that were used in the SEM analysis. Short explanations are given. In the second section we provide results that we could not include in the paper for readability considerations. Items from the CHLQ-A scales with descriptive statistics is displayed along with tables of residuals from all steps in the analysis. We also present two different results from the final model, on in which we specify a lower factor loading (λ = .70) for the indicator of PE than what was specified in the model presented in the paper, and one where the loading is higher (λ = 0.90).

1.1 Model syntax and commands

As reported in the paper we estimated the measurement model with covariance between latent variables. We used the following model syntax ('lavaan' automatically estimates covariance in multiple factor models if nothing else is specified). In the subsequent steps, with regressions, covariance between latent factors must be specified.

```
mod <-"
CHLC1 =~ chl36 + chl37 + chl38
CHLC2 =~ chl39 + chl40 + chl41
PE =~ 0.8*kro"
```

Step 1

```
mod1 <-"
# measurement model

CHLC1 =~ chl36 + chl37 + chl38
CHLC2 =~ chl39 + chl40 + chl41
PE =~ 0.8*kro"

# Regressions

CHLC1 + CHLC2 ~ PE

# Covariances

CHLC1 ~~ CHLC2
```

Step 2

```
mod2 <-"
# measurement model

CHLC1 =~ chl36 + chl37 + chl38
CHLC2 =~ chl39 + chl40 + chl41
PE =~ 0.8*kro"

# Regressions

CHLC1 + CHLC2 ~ PE + ParEd

PE ~ ParEd

# Covariances

CHLC1 ~~ CHLC2
```

Step 3

```
Mod3 <-"
# measurement model
```

```
CHLC1 =~ chl36 + chl37 + chl38
CHLC2 =~ chl39 + chl40 + chl41
PE =~ 0.8*kro"

# Regressions

CHLC1 + CHLC2 ~ PE + ParEd + PA

PE ~ ParEd + PA

# Covariances

CHLC1 ~~ CHLC2
```

Step 4

```
Mod4 <-"
# measurement model

CHLC1 =~ chl36 + chl37 + chl38
CHLC2 =~ chl39 + chl40 + chl41
PE =~ 0.8*kro"

# Regressions

CHLC1 + CHLC2 ~ PE + ParEd + PA + sport

PE ~ ParEd + PA + sport

# Covariances

CHLC1 ~~ CHLC2
```

1.2 Commands for model estimation

In the command we used to estimate the model we specified type of estimator (e.g. ULSMV) and that the data should be treated as ordinal. The std.lv command tells lavaan to restrict the variance of latent constructs to 1 instead of the factor loading of the first indicator of each factor (which is the default scaling). In this case we specified the factor loading of the indicator of the PE construct, and therefore had to standardize the left side of the equations (the variance of the latent construct).

```
fitmod <- sem(model = mod, data= df, estimator='ULSMV', ordered = TRUE,
std.lv = TRUE)
```

2. Supplementary Tables

In this section we present results that were not included in the paper. In the first section we present the items of CHLA-Q that were used with descriptive statistics. In the next section we show the residuals from the four steps of estimation in the SEM analysis. Lastly, we display the results from the final model with different factor loadings for PE.

2.1 Items and item statistics

The items from the CHLA-Q instrument that were used in this study with mean, kurtosis, variance, and skew.

Table S1. Items and item statistics of CHL-C1 and CHL-C2

Var	**I am a person who…** (1: completely incorrect; 2: incorrect; 3: sometimes correct; 4: correct; 5: completely correct)	**Mean (SD)**	**Kurtosis**	**Variance**	**Skew**
chl36	…can help others if they are not doing well.	4.14	1.05	.67	-.94
chl37	…can contribute to the well-being of others in my class.	3.88	.16	.68	-.45
chl38	…can help find solutions that are acceptable to all parties.	3.71	-.11	.70	-.23
chl39	…can easily talk to others, even if I don't know them very well.	3.35	-.87	1.53	-.31
chl40	…can share information about factors that influence health with others.	3.34	-.42	1.11	-.29
chl41	…believes my knowledge about health could be useful for others.	3.02	-.62	1.22	-.11

2.2 Residuals

Four tables that show the standardized residuals from each of the main steps of the analysis.

Table S2. Residuals from estimation step 1

	chl36	chl37	chl38	chl39	chl40	chl41	kro

chl36	0.000						
chl37	0.064	0.000					
chl38	-0.056	-0.009	0.000				
chl39	0.023	0.008	0.001	0.000			
chl40	0.005	-0.061	0.005	-0.004	0.000		
chl41	-0.019	-0.037	0.081	-0.048	0.046	0.000	
kro	-0.047	0.027	0.017	0.073	-0.007	-0.060	0.000

Table S3. **Residuals from estimation step 2**

Measurement model

	chl36	chl37	chl38	chl39	chl40	chl41	kro
**chl36**	0.000						
**chl37**	0.065	0.000					
**chl38**	-0.056	-0.010	0.000				
**chl39**	0.032	0.018	0.010	0.000			
**chl40**	0.003	-0.065	0.002	0.004	0.000		
**chl41**	-0.023	-0.042	0.078	-0.042	0.033	0.000	
**kro**	-0.048	0.027	0.017	0.086	-0.010	-0.066	0.000

Regression model

	ParEd
**chl36**	-0.011
**chl37**	0.008
**chl38**	0.002
**chl39**	-0.108
**chl40**	0.041
**chl41**	0.054
**kro**	0.000

Table S4. **Residuals from estimation step 3**

Measurement model

	chl36	chl37	chl38	chl39	chl40	chl41	kro
**chl36**	0.000						
**chl37**	0.056	0.000					
**chl38**	-0.046	-0.012	0.000				
**chl39**	0.023	-0.007	0.008	0.000			
**chl40**	0.008	-0.074	0.013	-0.007	0.000		
**chl41**	-0.012	-0.043	0.095	-0.046	0.048	0.000	
**kro**	-0.047	0.009	0.034	0.070	-0.008	-0.056	0.000

Regression model		
	ParEd	PA
chl36	-0.006	-0.031
chl37	-0.010	0.089
chl38	0.016	-0.065
chl39	-0.131	0.101
chl40	0.048	-0.020
chl41	0.071	-0.072
kro	0.000	0.000

Table S5. Standardized residuals from estimation step 4

Measurement model							
	chl36	chl37	chl38	chl39	chl40	chl41	kro
chl36	0.000						
chl37	0.065	0.000					
chl38	-0.056	-0.010	0.000				
chl39	0.032	0.018	0.010	0.000			
chl40	0.003	-0.065	0.002	0.004	0.000		
chl41	-0.023	-0.042	0.078	-0.042	0.033	0.000	
kro	-0.048	0.027	0.017	0.086	-0.010	-0.066	0.000

Regression model			
	ParEd	PA	Sport
chl36	-0.008	-0.051	0.040
chl37	-0.011	0.076	0.031
chl38	0.019	-0.032	-0.070
chl39	-0.138	0.035	0.149
chl40	0.049	0.007	-0.056
chl41	0.072	-0.038	-0.074
kro	0.000	0.000	0.000

2.3 Results from final model with different factor loadings for the latent construct PE

In this section we present the main results of models with slightly lower and higher factor loadings specified for the single-indicator latent construct of PE. We see that although there are some changes the overall results point in the same direction. If we reduce the factor loading (table S6) for the indicator of PE ($\lambda = 0.7$) the associations become slightly stronger, and the model explains more of the variance in the dependent variables. When the factor loading is higher ($\lambda = 0.9$) the pattern is opposite (table S7).

Table S6 Factor loading for the indicator of PE set to .70.

	Unstandardized		Standardized			
**Parameter**	**Estimate**	**SE**	**Estimate (β)**	**SE**	**P-value**	**R-squared**
**CHL-C1**						10.4 %
PE	.266	.079	.315	.088	.001	---
Parents ed.	.044	.115	.021	.053	.698	---
Leisure PA	.089	.138	.042	.065	.519	---
Sport clubs	-.089	.152	-.040	.068	.558	---
**CHL-C2**						15.7 %
PE	.365	.090	.419	.092	.000	---
Parents ed.	.094	.119	.042	.054	.430	---
Leisure PA	.121	.141	.056	.065	.389	---
Sport clubs	-.344	.158	-.149	.067	.030	---
**PE**						36.0 %
Parents ed.	.300	.149	.118	.058	.045	---
Leisure PA	.702	.165	.281	.063	.000	---
Sport clubs	.960	.175	.363	.061	.000	---

Standardized and unstandardized regression coefficients with standard errors (SE), p-value. R-squared are given for dependent variables.

Table S7 Factor loading for the indicator of PE set to .90.

	Unstandardized		Standardized			
**Parameter**	**Estimate**	**SE**	**Estimate (β)**	**SE**	**P-value**	**R-squared**
**CHL-C1**						7.9 %
PE	.204	.059	.227	.063	.001	---
Parents ed.	.075	.109	.035	.051	.492	---
Leisure PA	.160	.127	.077	.061	.206	---
Sport clubs	.012	.135	.005	.061	.931	---
**CHL-C2**						11.3 %
PE	.277	.065	.302	.066	.000	---

Parents ed.	.134	.110	.062	.051	.223	---
Leisure PA	.217	.127	.102	.059	.087	---
Sport clubs	-.201	.136	-.089	.060	.140	---
**PE**						25.4 %
Parents ed.	.233	.116	.099	.049	.045	---
Leisure PA	.546	.128	.236	.053	.000	---
Sport clubs	.747	.136	.304	.052	.000	---

Standardized and unstandardized regression coefficients with standard errors (SE), p-value. R-squared are given for dependent variables.
